# Characteristics of imitation Mozzarella cheese manufactured without emulsifying salts using a combination of culture‐based acid curd and micellar casein concentrate

**DOI:** 10.1002/fsn3.3424

**Published:** 2023-05-15

**Authors:** Ahmed R. A. Hammam, Lloyd E. Metzger

**Affiliations:** ^1^ Dairy and Food Science Department South Dakota State University Brookings South Dakota USA; ^2^ Dairy Science Department, Faculty of Agriculture Assiut University Assiut Egypt

**Keywords:** acid curd, functional characteristics, imitation Mozzarella cheese, melting properties, micellar casein concentrate

## Abstract

The objectives of this study were to develop a process to produce acid curd from micellar casein concentrate (MCC) using starter cultures and to manufacture imitation Mozzarella cheese (IMC) using a combination of acid curd and MCC that would confer emulsification ability to the caseins without the use of emulsifying salts (ES). The formulations were targeted to produce IMC with 49.0% moisture, 20.0% fat, 18.0% protein, and 1.5% salt. In the IMC formulation made without ES (FR‐2:1), the acid curd was blended with MCC so that the formula contained a 2:1 ratio of protein from acid curd relative to MCC. IMC with ES was also produced as a control. The melt and stretch characteristics of IMC made from FR‐2:1 were similar to those of control IMC. We conclude that IMC can be made without ES using a 2:1 ratio of protein from acid curd relative to MCC.

## Graphical abstract

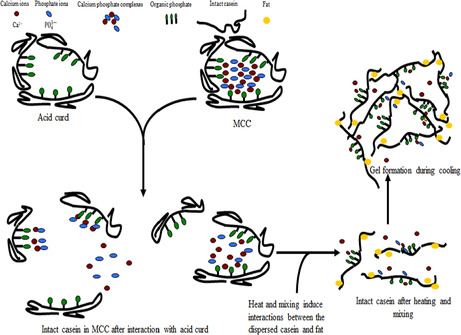

## 1 INTRODUCTION

Microfiltration (MF) is a membrane process that is utilized to fractionate casein (CN) and serum protein (SP) from skim milk using a 0.1 μm semipermeable membrane. The skim milk is force driven through the membrane to separate CN (retentate side) and SP (permeate side) based on their sizes (0.1–0.4 μm CN vs. 0.003–0.01 μm SP). The retentate is called micellar casein concentrate (MCC), which is mostly native casein with approximately 9.0% total protein (TPr) and 13.0% total solids (TS). Micellar casein concentrate is a high protein ingredient that is typically manufactured in 3 MF stages using a 3× concentration factor (CF) with diafiltration (DF).

Micellar casein concentrate has promising applications in some dairy and nondairy products due to its unique physicochemical and functional characteristics (e.g., foaming, emulsifying, wetting, dispersibility, heat stability, and water‐binding ability). The high casein content in MCC makes it heat stable and thereby it can be used in beverages that require sterilization (Beliciu et al., 2012; Sauer & Moraru, 2012). The nondairy applications of MCC are pasta, confectionery, meat products, special dietary preparations, and convenience foods (Hammam et al., 2021; Salunke, 2013). The dairy applications for MCC include Cheddar cheese (Amelia et al., 2013; Li et al., 2020; Xia et al., 2021), Feta cheese (Hammam, Kapoor, et al., 2022), Greek‐style yogurt (Bong & Moraru, 2014), imitation Mozzarella cheese (IMC) (Salunke et al., 2022), process cheese (PC), process cheese products (PCP) (Hammam, Beckman, et al., 2022; Metzger & Hammam, 2020; Salunke & Metzger, 2022), and acid curd (Hammam et al., 2023; Metzger & Hammam, 2020). We recently developed a process to produce soft acid curd from MCC using direct acidification (lactic acid) (Hammam et al., 2023; Metzger & Hammam, 2020).

The IMC is a dairy, partial dairy, or nondairy food depending on the sources of protein and fat used in the formulation. The typical protein source for IMC is rennet casein and fat sources can range from milk fat to vegetable oils/fats depending on the final nutritional, functional, and cost targets. This type of cheese is one of the popular analogue cheeses in the United States due to its applications in pizza (Bachmann, 2001; O'Riordan et al., 2011). It also offers manufacturers the flexibility to produce a final product with desired characteristics (shreddability, meltability, flowability, stretchability, chewiness, oiling off, and browning on baking) that are more consistent over a longer shelf‐life when compared to natural cheese (Bachmann, 2001; Guinee et al., 1999; O'Riordan et al., 2011). The meltability and stretchability characteristics are important in IMC due to the application of this type of cheese in pizza.

The IMC has the same basic principles that are used in the manufacture of PC in terms of process and equipment. It is prepared by blending dairy (rennet casein and milk permeate) and nondairy ingredients (edible oils/fat, protein, and emulsifying salts: ES, NaCl, acidulant, and water) with the aid of heat and shear to produce a homogeneous product (Hammam, Beckman, et al., 2022; Metzger & Hammam, 2020; O'Riordan et al., 2011). A critical reaction that occurs during IMC or PC manufacture is calcium sequestration using ES. The ES such as trisodium citrate and disodium phosphate are critical for the functional characteristics of IMC due to their role in improving the emulsification characteristics of casein by sequestering a portion of the calcium from the calcium–casein–phosphate network. As a result, the major molecular forces that cross‐link the various monomers of casein are partially disrupted by the sequestered calcium complexes. This disruption leads to hydration and dispersion of the protein. The partially dispersed monomers of casein have hydrophilic and hydrophobic portions that have emulsification properties. This, in turn, links the hydrophilic aqueous phase with the hydrophobic fat phase (Guinee et al., 2004), which prevents oil separation in IMC. In the presence of heating and mixing, a homogeneous IMC is produced.

**FIGURE 1:**
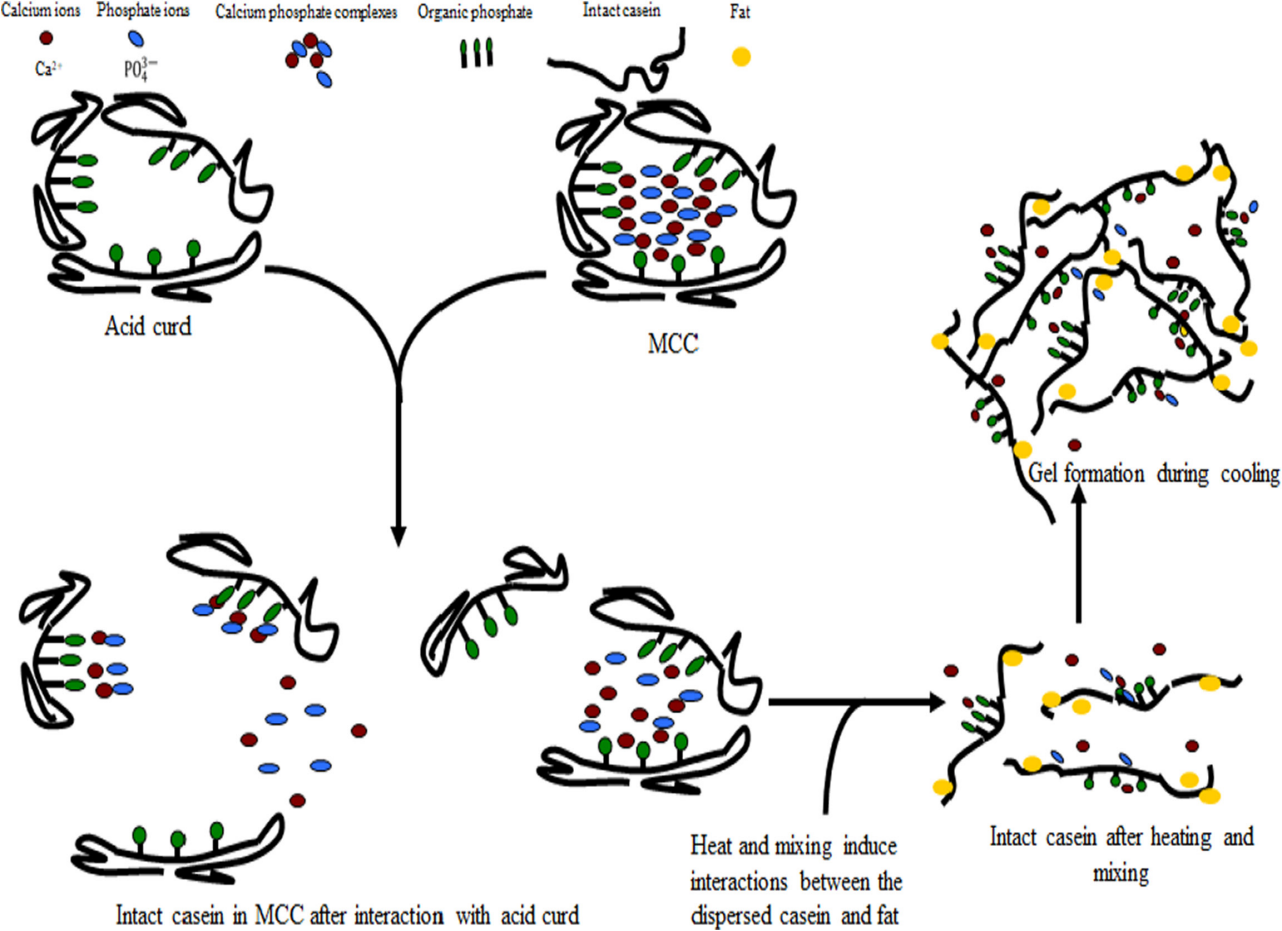
Acid curd and micellar casein concentrate (MCC) interaction in making imitation Mozzarella. Adapted (Hammam et al., 2023).

Acid curd is a protein concentrate (>80% total protein as a percentage of total solids), which can be obtained by precipitating casein at pH 4.6 (isoelectric point) using starter cultures or acids without the use of rennet. The colloidal calcium phosphate in the micelles is dissolved in the whey at a pH of 4.6, and this results in acid curd with low mineral or calcium content. In contrast to the low mineral content in acid curd, MCC has a high level of casein‐bound calcium with a pH of 6.5–6.7. If acid curd is mixed with MCC (Figure 1), it may be possible to create a partially deaggregated casein network without the use of ES. The ratio of acid curd to MCC will have an impact on the level of deaggregation and the pH of the final product. In our previous patent, we hypothesized that a ratio of two parts of protein from acid curd to one part of protein from MCC created a partially deaggregated casein network similar to a typical PC that utilizes ES (Figure 1) (Metzger & Hammam, 2020), and we hypothesized that this can also occur in IMC formulations.

Acid curd can be produced from skim milk in a process similar to Cottage cheese manufacture. There is a possibility of using MCC instead of skim milk to produce acid curd. Making acid curd from MCC has advantages as compared to skim milk, since manufacturing MCC using MF results in milk‐derived whey protein as a by‐product which can be utilized in many value‐added applications, particularly making whey protein isolate. In contrast, acid curd produced from skim milk results in acid whey as a by‐product, which is more challenging to utilize. The typical composition of MCC (three stages using 3× CF with DF) is >9% true protein (TP) and >13% TS (Zulewska et al., 2009). This MCC could be used immediately in making acid curd or diluted to lower protein levels before making acid curd if required. In our previous studies, we produced acid curd from MCC with different protein content (3%, 6%, and 9%). We found that MCC with 9% protein is the optimum ingredient to produce acid curd (pH = 4.6) using lactic acid (Hammam et al., 2023; Metzger & Hammam, 2020). Although direct acids such as lactic acid take less time to produce the acid curd, those acids are costly and produce acid curd with a bland flavor compared to the varieties of starter cultures that have less cost and can be utilized to develop flavors in the curd.

Many consumers are perceiving ES as chemicals which are reducing the consumption of products like IMC. As a result, manufacture of IMC with no ES would meet the consumer desire. Therefore, the objectives of this study were to develop a process to produce acid curd from MCC (~9% TP and ~13% TS) using starter cultures and to determine if IMC could be produced in the absence of ES using a combination of acid curd and MCC in the formulations.

## 2 MATERIALS AND METHODS

### 2.1 Preparation of MCC solution

**FIGURE 2:**
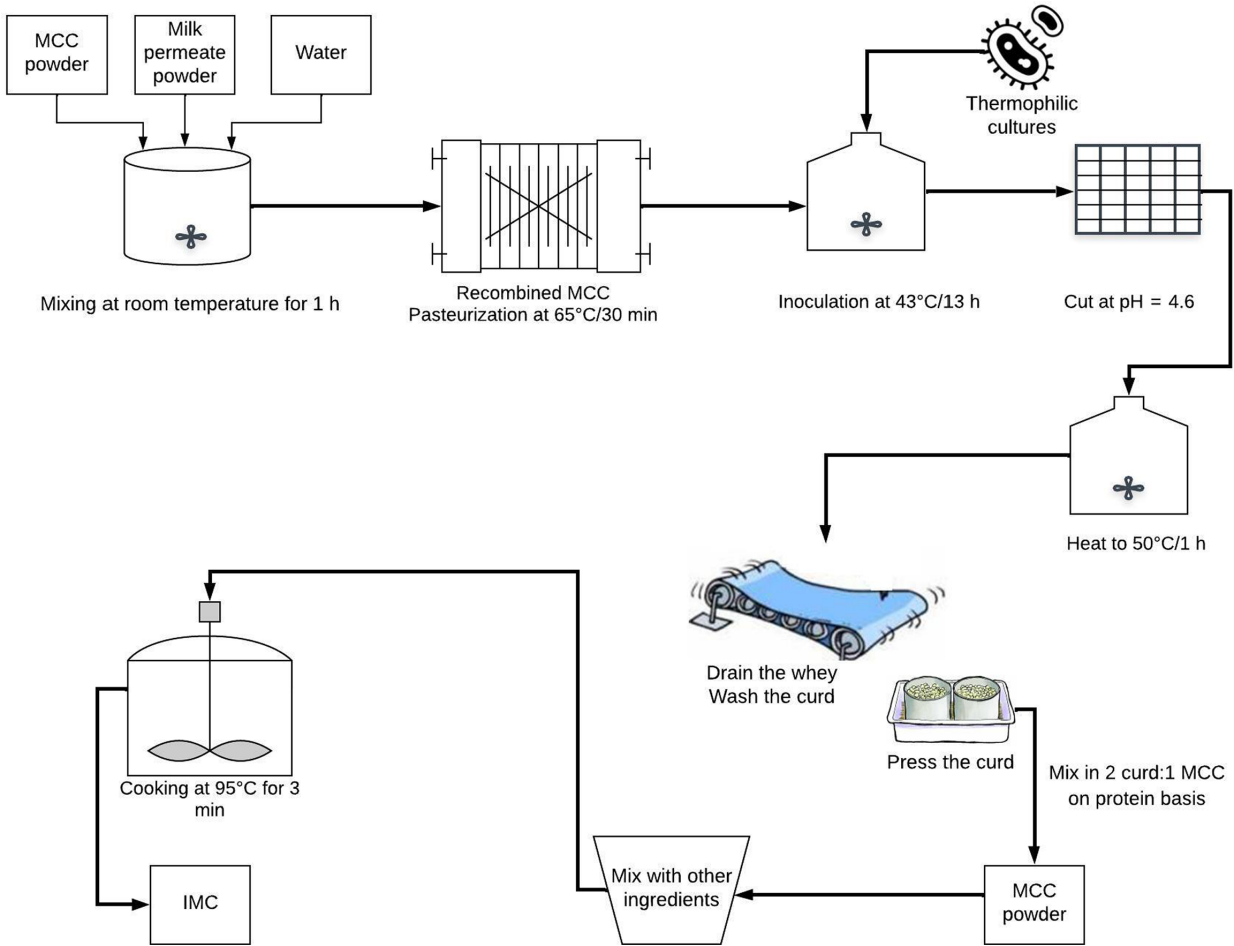
Schematic diagram for manufacturing of recombined micellar casein concentrate (MCC), acid curd, and imitation Mozzarella cheese (IMC).

The MCC solution (pH ~6.6) was prepared (Figure 2) and standardized by mixing MCC powder (CasPro 8500, Lot # NF8109A1, Milk Specialties Global, Eden Prairie, MN 55344), milk permeate powder (product lot: 19113D40, Idaho Milk Products, ID), and water to produce a recombined MCC with an average of 13.0% TS, 9.0% TPr, and 2.0% lactose (the minimum lactose amount that is required to be fermented by the starter cultures to get the pH of 4.6). Techwizard software (Excel‐based formulation software program provided by Owl Software) was used to prepare 1 L of the recombined MCC. Powder ingredients were mixed with water using a magnetic stirring plate for 1 h at room temperature and then batch pasteurized at 65°C for 30 min.

### 2.2 Manufacture of culture‐based acid curd

Thermophilic cultures (i455, Batch no 3489654, Chr Hansen) were added at a rate of 0.005% to the recombined MCC and incubated at 43°C (Major Science, Saratoga, CA 95070, USA) for about 15 h to decrease the pH to 4.6 (Figure 2). After reaching the pH of 4.6, the curd was cut and stirred gently during heating to 50°C for 1 h. The whey was subsequently drained, and the curd was washed with water at a 1:1 ratio, pressed, and kept in the freezer for further analyses. This trial was repeated three times.

### 2.3 Manufacture of imitation Mozzarella cheese

Techwizard was also used to develop the IMC formulations (Metzger & Roland, 2017) to produce IMC with 49.0% moisture, 20.0% fat, 18.0% protein, and 1.5% salt. The percentage of ingredients utilized in IMC formulations is shown in Table 1. In the formulation of 2:1 (FR‐2:1), the amount of protein from acid curd and MCC in the mixture was adjusted to have a ratio of 2:1, respectively. The ingredients of FR‐2:1 included salt (NaCl, Morton salt, INC, Chicago, IL), water, commercial vegetable oil (Wesson, Omaha, NE), MCC powder, milk permeate, and acid curd. Control IMC formulation (Table 1) was made using rennet casein (Rennet Casein 90 Mesh, Fonterra INC, Rosemont, IL) with NaCl salt, water, commercial vegetable oil, milk permeate, citric acid (KIC chemical Inc, New Peltz, NY), and Kasal salt as ES (chelating salt blend and sodium aluminum phosphate basic powder, Innophos, Chicago Height, IL). The milk permeate was used to standardize the solids content to 51.0% in the formulations. The formulations were prepared in a 300.0 g mix by mixing all the ingredients in a KitchenAid at room temperature for 30 min to produce a homogeneous paste. A sample (20 g) of the paste was weighed in a canister and then cooked in the Rapid Visco Analyzer (RVA; Perten RVA 4500) for 3 min at 95°C (Figure 2). Water (0.5 g) was added to each canister to compensate for the water that evaporated during mixing and cooking in RVA. The sample was stirred for 2 min at 1000 rpm and subsequently at 160 rpm for 1 min. Ten canisters from each batch were cooked in the RVA. The canisters were poured into plastic molds (diameter = 28.3 mm; height = 25 mm) to measure the melt temperature using dynamic stress rheometry (DSR) and melt diameter using the Schreiber melt test.

**TABLE 1:** Imitation Mozzarella cheese (IMC) formulations made with acid curd and micellar casein concentrate (MCC).

Ingredients	Treatment (%)^a^	
	Control	FR‐2:1
Salt	1.50	1.50
Water	46.44	11.56
Vegetable oil, canola	19.98	19.89
MCC powder	0.00	7.19
Acid curd	0.00	49.77
Rennet casein	21.05	0.00
Milk permeate powder	8.73	10.09
Citric acid	0.90	0.00
Kasal salt (sodium aluminum phosphate)	1.40	0.00
Total	100.0	100.0

^a^ Treatment: Control = IMC made with rennet casein and emulsifying salts (ES); FR‐2:1 = IMC made with no ES using a 2:1 ratio of protein from acid curd to MCC.

### 2.4 Chemical analyses

The acid curd was analyzed for TS (AOAC, 2000), TPr = total nitrogen × 6.38 (AOAC, 2000), ash (AOAC, 2000), lactose along with lactic acid (Amamcharla & Metzger, 2011; González de Llano et al., 1996), and mineral profile (AOAC, 2000). The whey (a by‐product of making curd) was analyzed for TS, TPr, ash, lactose, and lactic acid. The TS and pH (Hannah Edge Blu, Woonsocket, RI) of the final IMC were also determined.

### 2.5 Functional analyses

#### 2.5.1 The end apparent cooked viscosity

The end apparent cooked viscosity was measured following the same procedures we performed in our previous studies (Hammam, Beckman, et al., 2022). The end apparent cooked viscosity of the IMC was measured by the end of the cooking time using the RVA at 95°C by calculating the mean value of the last five values, which is referred to as the end apparent viscosity. The end apparent cooked viscosity was measured in all canisters of each batch.

#### 2.5.2 Dynamic stress rheometry (DSR)

The DSR was performed following previous studies (Hammam, Beckman, et al., 2022). The IMC sample was prepared by cutting the cheese into slices (2 mm thick and 28.3 mm diameter) using a wire cutter. A stress sweep test of the IMC was performed at a frequency of 1.5 Hz, and stress ranged from 1 to 1000 Pa at 20°C using a rheometer with parallel plate geometry (MSR 92, Anton Paar, Graz, Austria). The stress sweep experiment revealed that the maximum stress limit for the linear viscoelastic region was 500 Pa. The dynamic rheological properties of the IMC were then analyzed with a dynamic temperature ramp test that ranged from 20 to 90°C with a ramp rate of 1°C/min using a frequency of 1.5 Hz and constant stress of 500 Pa. The temperature at which tan δ = 1 (G″/G′) was referred to as the cheese melting temperature. A duplicate test was performed on each batch.

#### 2.5.3 Schreiber melt test

The IMC samples were cut into cylinders (diameter = 28.3 mm and height = 7 mm) and placed in Petri dishes. The dishes were transferred to a forced draft oven at 90°C for 7 min (Hammam et al., 2023; Muthukumarappan et al., 1999). After cooling the dishes to room temperature, the diameter of the melted IMC samples was measured in four different places using a Vernier caliper and reported in millimeters. The melting area (A) was calculated using the radius I of the cheese (A = *π r* ^2^). This test was repeated four times for each batch.

#### 2.5.4 Stretchability test

The stretchability of IMC samples was measured using the method described by (Gunasekaran & Ak, 2003) with some modifications. The test was done by placing a cylinder of cheese (diameter = 28.3 mm and height = 7 mm) in a glass Petri dish (95.0 mm diameter) and left in a forced draft oven at 232°C for 3 min and 30 s (Muthukumarappan et al., 1999). It was cooled for 30 s and then a four‐pronged fork was inserted into the cheese. Subsequently, the fork was lifted vertically and the distance before breaking the cheese was measured in centimeters. This test was replicated four times.

### 2.6 Statistical analysis

Statistical analysis of the data generated was performed to study the effect of formulations on the functional properties of IMC. An ANOVA was done using R software (R × 64–3.3.3, R Foundation for Statistical Computing, Vienna, Austria). Differences were tested using the least significant difference test at *p* < .05.

## 3 RESULTS AND DISCUSSION

### 3.1 Composition of acid curd and acid whey

The composition of acid curd produced from MCC is shown in Table 2. The mean composition of acid curd from three replicates was 25.8% TS, 23.7% protein, 0.9% ash, 0.2% lactose, 0.6% lactic acid, 0.2% Ca, and 0.1% P. The average pH of acid curd was targeted to have 4.6. The composition of acid curd depends on the composition of initial materials, final pH, and process conditions (e.g., cooking temperature, washing curds, and pressing) (Wong et al., 1976). The step of washing the curd could decrease the ash, Ca, P, lactose, and lactic acid content in the final acid curd. Since the lactose is converted into lactic acid using starter cultures as a result of fermentation, lactose decreased while lactic acid increased as the pH reached 4.6. It is also expected that the ash content would decrease when the reconstituted MCC solution is converted into acid curd (Hill et al., 1985; Lucey & Fox, 1993; Wong et al., 1976). The Ca is converted from insoluble (colloidal form) to soluble form and released in the whey as the pH decreases (Dalgleish & Law, 1989; Guinee et al., 1993; Wong et al., 1976). As a result, the Ca and P were reduced as the pH decreased yielding a low ratio of Ca to P (Kindstedt & Kosikowski, 1988; Lucey & Fox, 1993), which results in low ash content.

**TABLE 2:** Composition (% by weight) of acid curd and acid whey produced from fermentation recombined micellar casein concentrate (MCC).

Ingredients	Composition (%)^a^						
	TS	TPr	Ash	Lactose	Lactic acid	Ca	P
Acid curd	25.81 ± 1.73	23.70 ± 1.30	0.89 ± 0.04	0.21 ± 0.02	0.63 ± 0.05	0.19 ± 0.01	0.14 ± 0.02
Acid whey	5.01 ± 0.07	1.42 ± 0.03	0.88 ± 0.02	0.58 ± 0.03	1.44 ± 0.03		

^a^ TS = total solids; TPr = total protein = total nitrogen × 6.38; Ca = calcium; P = phosphate.

The composition of acid whey (a by‐product of making the curd from MCC) is illustrated in Table 2. The acid whey produced as a by‐product during making the curd showed an average of 5.0% TS, 1.4% protein, 0.9% ash, 0.6% lactose, and 1.4% lactic acid. The total protein as a percentage of total solids in acid whey produced on the lab scale was 28.0%. Results in Table 2 showed that approximately 1.4% of lactose was required to reach a pH of 4.6 in the acid curd/whey. Using 2.0% lactose MCC left around 0.6% of lactose in the lab scale acid whey. The loss of components in whey, especially protein, depends on the composition of the initial material as well as handling the curd in the cheese vat. The composition of acid whey produced as a by‐product of making acid curd using MCC was similar to the acid whey produced from milk in other studies (Chandrapala et al., 2015; Chen et al., 2016; Saffari & Langrish, 2014). Those studies found that the TS of acid whey ranged from 5.0% to 7.0%, while protein and ash ranged from 0.5 to 1.0 and 0.5% to 1.0%, respectively. The protein in acid whey could increase to 1.4% as in our study with elevating the protein content in MCC (9.2% protein), which was expected. As a result, solids, ash, and protein content in acid whey can be changed based on the composition of starting material. The lactose content in acid whey was different compared to other studies since we started with 2.0% lactose MCC, not 4.5% lactose as in milk.

### 3.2 Composition of IMC

The moisture content and pH of IMC are shown in Table 3. The IMC formulations were targeted to have 49.0% moisture. The actual moisture content was approximately 48.3% in IMC made with ES (control) and 48.2% in IMC made with no ES (FR‐2:1 formulation). No significant differences (*p* > .05) were detected in the moisture content of IMC between the control and FR‐2:1. It was expected to have some moisture loss during mixing and cooking the IMC. It was found in previous studies that there is a loss of evaporated water during cooking PC and IMC (Hammam, Beckman, et al., 2022; Kommineni et al., 2012; Salunke, 2013). However, we added 0.5 g of water into each canister (20 g) to compensate for the evaporated water from the cooked cheese (Purna et al., 2006).

**TABLE 3:** Moisture and pH of the imitation Mozzarella cheese (IMC).

Treatment^1^	Moisture (%)	pH
Control	48.29 ± 0.37	5.72 ± 0.01^a^
FR‐2:1	48.21 ± 0.07	5.37 ± 0.03^b^
SEM	0.09	0.08

*Note*: Means in the same column not sharing a common superscript (^a,b^) are different at *p* < .05.

Abbreviation: SEM, Standard error of the mean.

^1^ Treatment: Control = IMC made with rennet casein and emulsifying salts (ES); FR‐2:1 = IMC made with no ES using a 2:1 ratio of protein from acid curd to MCC.

The pH of the control IMC was 5.7, while the pH of the experimental IMC made from FR‐2:1 was 5.4. Significant differences (*p* < .05) were detected in the pH of control and FR‐2:1 IMC. We previously manufactured PCP with no ES using acid curd and MCC at the same ratio. The pH of the PCP was 5.4 (Hammam et al., 2023; Metzger & Hammam, 2020). The main roles of using ES in PC formulations are calcium sequestration and pH adjustment (Kapoor & Metzger, 2008; Shirashoji et al., 2010). ES sequesters the Ca from the casein network to produce a deaggregated casein network. ES exerted buffering action which could affect the amount of casein‐bound calcium and thereby the pH of final PC (Brickley et al., 2008; Shirashoji et al., 2010). This might affect the characteristics of the final PC, as a result, the type of ES and typical amounts should be considered to have the desired PC (Kapoor & Metzger, 2008). The IMC made from FR‐2:1 had low pH compared to control. It was found that the pH of IMC can range from 5.4 to 5.8 when ES was utilized (Bi et al., 2016; Jana et al., 2010; Noronha et al., 2008), which is similar to the typical pH of PC or PCP (Bulut‐Solak & Akin, 2019; Kapoor & Metzger, 2008; Marchesseau et al., 1997). PC or PCP and IMC have the same basic principles and the same interactions during cooking of the cheese. Palmer and Sly stated that the emulsion stability of PC is poor when the pH is lower than 5.4 or higher than 5.8 (Palmer & Sly, 1943). The differences in pH of control IMC made with ES relative to IMC made from FR‐2:1 could affect the structure and quality of final IMC and thereby its functional properties due to its effects on the protein interactions in the final IMC emulsion (Marchesseau et al., 1997; Meyer, 1973; Palmer & Sly, 1943). As the pH of PC was reduced to 5.2, the protein–protein interaction increased (Marchesseau et al., 1997) because this pH is close to the isoelectric point of caseins (4.6). This induced the aggregation of protein, which, in turn, resulted in a poor emulsion of fat in IMC. On the other hand, the PC had an open structure when the pH was elevated to 6.1, which eventually led to a weaker emulsification (Marchesseau et al., 1997). Marchesseau also found that the pH of 5.7 resulted in PC with more uniform fat emulsion with a closely knit protein network (Marchesseau et al., 1997).

### 3.3 Functional characteristics of IMC

The end apparent cooked viscosity of IMC is presented in Table 4. The end apparent cooked viscosity is referred to as the cheese's flowability when completely melted, which is measured at the end of cooking time (Prow & Metzger, 2005). The end apparent cooked viscosity of IMC was around 5711.0 and 7500.0 cP for IMC made from control and FR‐2:1, respectively. Although the 2:1 IMC showed higher cooked viscosity relative to control IMC, no significant differences (*p* > .05) were detected.

**TABLE 4:** The functional properties of the imitation Mozzarella cheese (IMC).

Treatment^1^	End apparent cooked viscosity (cP)	Melt temperature (°C)	Melt diameter (mm)	Melt area (mm^2^)	Stretchability (cm)
Control	5711.33 ± 33.63	55.54 ± 6.33	29.41 ± 0.07^b^	679.60 ± 3.45^b^	12.50 ± 1.00
FR‐2:1	7499.59 ± 810.01	49.96 ± 2.64	31.57 ± 0.67^a^	783.10 ± 33.09^a^	12.30 ± 0.12
SEM	451.34	2.16	0.51	24.68	0.26

*Note*: Means in the same column not sharing a common superscript (^a,b^) are different at *p* < .05.

Abbreviation: SEM, Standard error of the mean.

^1^ Treatment: Control = IMC made with rennet casein and emulsifying salts (ES); FR‐2:1 = IMC made with no ES using a 2:1 ratio of protein from acid curd to MCC.

The melting characteristics of IMC are shown in Table 4. The melt temperature of control was 55.5°C relative to 50.0°C in IMC made with no ES. The melt temperature of IMC made from control was not significantly different (*p* > .05) as compared to that of the FR‐2:1 formulation. The melting diameter and melt area were 29.4 mm and 679.6 mm^2^, respectively, in IMC made with ES; while they were 31.6 mm and 783.1 mm^2^ in IMC made from FR‐2:1. The melting diameter and melt area were significantly (*p* < .05) lower in control IMC compared to those in IMC made from FR‐2:1. However, control IMC was as stretchable (*p* > .05) as IMC made with no ES. The stretchability was 12.5 and 12.3 cm in control and FR‐2:1 formulation, respectively.

Noronha found that the melting temperature of IMC (approximately 48.0% moisture, 23.0% protein, 25.0% fat, and 5.9 pH) ranged from 65.3 to 69.5°C (Noronha et al., 2008). Salunke made IMC (48.0% moisture, 20.0% protein, 21.0% fat, and 5.8 pH) from MCC and MPC individually using about 25.0% of those ingredients in the formulations (Salunke, 2013). That study reported that the melting temperature of IMC made from MCC and MPC ranged from 70.0 to 71.0°C compared to 73.7°C in rennet casein IMC in the presence of ES. Salunke also found that the melted diameter and stretching on pizza were 31.81–32.82 mm (area of 795.0–846.0 mm^2^) and 0.6–1.4 cm (6.0–14.0 mm), respectively, in IMC made with MCC and MPC, while it was 37.56 mm (area of 1108.2 mm^2^) and 25.6 mm (area of 514.7 mm^2^), respectively, in IMC made with rennet casein (Salunke, 2013). The stretching of IMC (44.3% moisture, 21.8% fat, and 6.6 pH) reported in another study was 34.0–45.0 cm (Shah et al., 2010) using dairy cream as the source of fat and rennet casein as the source of protein.

The slight differences in the onset of melting (melting temperature) can be explained by the differences in pH of IMC. As the pH drops to the isoelectric point, the net negative charges on caseins reduce which increases the protein–protein interactions and this led to aggregation of protein and thereby poor emulsification (Kapoor, 2007). The higher pH in control IMC resulted in a uniform fat emulsion with a closely knit protein network. This led to a higher melting temperature of IMC made with ES relative to that of IMC made from FR‐2:1. As the pH elevated, the net negative charges on casein micelles increased, which promoted the calcium‐mediated cross‐links casein molecules, and this, in turn, strengthened the IMC gel network. Increasing the strength of IMC gel network led to restricting the movement of casein chains in IMC during reheating, which decreased the flowability, melt diameter, and melt area of IMC with higher pH made with ES (Kapoor, 2007). This phenomenon was not pronounced in the melting temperature and stretchability of IMC, but it was more differentiating in the melt diameter and melt area.

## 4 CONCLUSIONS

Culture‐based acid curd was produced from liquid MCC (>9% TPr and >13% TS). Acid curd and MCC can be mixed in a specific ratio and marketed to be ready for making different types of cheeses including IMC. Many consumers are perceiving ES as chemicals that are reducing the consumption of IMC. As a result, manufacture of IMC with no ES would meet consumers' desire. The MCC and acid curd were utilized successfully in manufacture of IMC with no ES in the ratio of 2:1 protein from acid curd to protein from MCC. This ratio of acid curd and MCC creates a partially deaggregated casein network that results in IMC with similar functionality to IMC produced with ES. IMC with no ES could be produced with similar functional characteristics to IMC made with ES.

## AUTHOR CONTRIBUTIONS

**Ahmed R. A. Hammam:** Conceptualization (equal); data curation (equal); formal analysis (equal); funding acquisition (equal); investigation (equal); methodology (equal); project administration (equal); resources (equal); software (equal); supervision (equal); validation (equal); visualization (equal); writing – original draft (equal); writing – review and editing (equal). **Lloyd E. Metzger:** Conceptualization (equal); data curation (equal); formal analysis (equal); funding acquisition (equal); investigation (equal); methodology (equal); project administration (equal); resources (equal); software (equal); supervision (equal); validation (equal); visualization (equal); writing – original draft (equal); writing – review and editing (equal).

## Data Availability

Research data are not shared.
